# Acute Supplementation with Nitrate-Rich Beetroot Juice Causes a Greater Increase in Plasma Nitrite and Reduction in Blood Pressure of Older Compared to Younger Adults

**DOI:** 10.3390/nu11071683

**Published:** 2019-07-22

**Authors:** Luke Stanaway, Kay Rutherfurd-Markwick, Rachel Page, Marie Wong, Wannita Jirangrat, Koon Hoong Teh, Ajmol Ali

**Affiliations:** 1 School of Sport, Exercise and Nutrition, Massey University, Auckland 0632, New Zealand; 2 School of Health Sciences, Massey University, Auckland 0632, New Zealand; 3 Centre for Metabolic Health Research, Massey University, Auckland 0745, New Zealand; 4 School of Health Sciences, Massey University, Wellington 6021, New Zealand; 5 School of Food & Advanced Technology, Massey University, Auckland 0632, New Zealand; 6 Thai Union Group PCL, Bangkok 10400, Thailand

**Keywords:** nitric oxide, beetroot juice, blood pressure, cognition, aging

## Abstract

Nitrate-rich beetroot juice supplementation has been shown to improve cardiovascular and cognitive function in younger and older adults via increased nitric oxide production. However, it is unclear whether the level of effects differs between the two groups. We hypothesized that acute supplementation with nitrate-rich beetroot juice would improve cardiovascular and cognitive function in older and younger adults, with the potential for greater improvements in older adults. Thirteen younger (18–30 years) and 11 older (50–70 years) adults consumed either 150 mL of nitrate-rich beetroot juice (BR; 10.5 mmol nitrate) or placebo (PL; 1 mmol nitrate) in a double-blind, crossover design, 2.25 h prior to a 30-min treadmill walk. Plasma nitrate and nitrite concentrations, blood pressure (BP), heart rate (HR), cognitive function, mood and perceptual tests were performed throughout the trial. BR consumption significantly increased plasma nitrate (*p* < 0.001) and nitrite (*p* = 0.003) concentrations and reduced systolic BP (*p* < 0.001) in both age groups and reduced diastolic BP (*p* = 0.013) in older adults. Older adults showed a greater elevation in plasma nitrite (*p* = 0.038) and a greater reduction in diastolic BP (*p* = 0.005) following BR consumption than younger adults. Reaction time was improved in the Stroop test following BR supplementation for both groups (*p* = 0.045). Acute BR supplementation increased plasma nitrite concentrations and reduced diastolic BP to a greater degree in older adults; whilst systolic BP was reduced in both older and younger adults, suggesting nitrate-rich BR may improve cardiovascular health, particularly in older adults due to the greater benefits from reductions in diastolic BP.

## 1. Introduction

The world’s population is aging, with the number of people aged over 60 years expected to increase from 901 million to 1.4 billion by 2030 [1]. Rates of disease and age-related dysfunction are increasing rapidly [2] and are expected to cause a dramatic increase in costs in the health and disability services areas [1]. This has led to an increased interest in the use of food-based supplements and bioactive compounds to improve or maintain health and body functions [3].

Beetroot juice contains high levels of nitrate (NO_3_^−^) which can be converted into the bioactive form, nitric oxide (NO) [4]. Nitric oxide plays a major role in many signaling pathways and biological processes, including improving neurotransmission and blood flow, alterations in mitochondrial oxygen consumption, promotion of cognitive benefits, mood and cardiovascular function [2,4]. Currently, two major pathways are known to result in the generation of NO in humans. First, through the conversion of l-arginine to NO by the enzyme nitric oxide synthase (NOS), which is considered the dominant pathway. Secondly, via the reduction of dietary NO_3_^−^ to NO_2_^−^ (nitrite), which occurs in the mouth by lingual anaerobic bacteria [4]. Nitrite is then swallowed and ultimately enters the bloodstream where it can be reduced further to NO [5]. It is through this second, exogenous pathway of NO production that nitrate-based food supplements such as BR are likely to exert their potential health benefits [4,6]. 

Acute supplementation with BR can improve physiological responses such as cardiovascular function in younger adults [7,8,9]. Specifically, consumption of inorganic NO_3_^−^, either in the form of sodium nitrate or BR, has been shown to result in a significant increase in plasma NO_2_^−^ levels and reduction of blood pressure in both younger (8 and 6 mmHg; systolic and diastolic blood pressure; respectively) [10] and older [4] (5 and 3 mmHg; systolic and diastolic blood pressure; respectively) adults. This positive effect on blood pressure has led to the suggestion that beetroot could potentially be used in medical settings as an alternative to traditional blood pressure-lowering drugs [11]. 

Acute supplementation with BR (7.5 mmol nitrate) has been shown to improve simple reaction times in older adults [12]. This effect may be due to an increase in blood flow to the brain which has been observed in older adults following consumption of dietary NO_3_^−^ [13]. Cognitive diseases have been linked to age-related reductions in cerebral blood flow (CBF) therefore nitrate supplementation may slow the process of age-related cognitive decline due to the aforementioned increased perfusion to the brain [13,14]. However, other studies have shown no improvement in cognitive function or mood with acute or 3 days of NO_3_^−^ supplementation in older adults [4,6]. The equivocal findings are likely due to methodological differences in the cognitive tests used, the duration of supplementation (from 2 h to 14 days), and amount of NO_3_^−^ consumed (6–12 mmol NO_3_^−^). 

Aging leads to a decline in body processes and functions resulting in increased blood pressure, reduced blood flow, and reduced oxygen delivery to the muscle. All of these measures have been shown to be improved following BR supplementation in both younger and older adults, however, data presented for older adults is limited [4,5]. There may be greater potential for improvement in cardiovascular and cognitive function, and mood following NO_3_^−^ supplementation in older adults relative to younger adults due to age-related changes [15]. Few studies have examined the health benefits of BR supplementation in older adults, and, to our knowledge, only one study directly compared the effects in younger versus older adults [16], therefore further research in this area is required. 

We hypothesized that acute supplementation with nitrate-rich beetroot juice would provide a greater improvement in cardiovascular responses, cognition, mood and perception in older adults (50–70 years) compared to younger adults (18–30 years). To examine this hypothesis we conducted a randomized, double-blind crossover trial to investigate and compare the effects of acute supplementation with nitrate-rich beetroot juice on plasma nitrate and nitrite concentrations, blood pressure and cognitive performance measures in older and younger adults.

## 2. Materials and Methods 

### 2.1. Sample Size

A power analysis was conducted (G-Power 3.1) to calculate the sample size based on the primary outcome measure of blood pressure. Previous literature in healthy adults has shown a mean change in BP of 10 mmHg (9 SD) between BR (9.6 mmol nitrate) and control [4]. Based on this, the required sample size was 11 per group, using an SD of 9, with a statistical power of 0.82 and α-level set at 0.05. 

### 2.2. Participants

Twenty-four healthy, normotensive, recreationally active adults (13 younger, 18–30 years, and 11 older, 50–70 years) volunteered for this study. Participants completed a health-screening questionnaire to ensure suitability. We did not recruit tobacco smokers, users of nitrate-based dietary supplements or well-trained/elite athletes (defined as those completing >4 training sessions per week, or >6 h of intense exercise per week, or having played/playing for a national or age-equivalent sports team). Those with known diseases (e.g., dementia and cardiovascular disease) were also excluded. Prior to testing, all participants were informed of the procedures, associated risks, and the possible benefits of participation, after which written informed consent was obtained. The study was approved by the Massey University Human Ethics Committee (SOA 16/27) and registered with ACTRN (ACTRN12618001466235).

### 2.3. Intervention 

Beetroot juice, extracted from fresh washed beetroots (beta vulgaris ‘Pablo’), was blended with other fruit juices to provide a standardized beetroot juice drink with a constant soluble solid concentration (11° Brix) and nitrate concentration (10.5 mmol/150 mL). The placebo beetroot drink was produced using beetroot juice concentrate and standardized to the same constant soluble solids concentration (11° Brix) but contained a low nitrate concentration (1 mmol/150 mL). The BR and placebo juice drinks had previously been presented to a consumer sensory panel using a triangle test, with 25 participants, and were not perceived as significantly different (*p* > 0.05). The nitrate concentration was analyzed using high-performance liquid chromatography (HPLC) based on the method of Cheng and Tsang [17]. Briefly, isocratic separation was achieved using a Gracesmart C-18 column; mobile phase 0.01 M Octylammonium orthophosphate (pH 3–3.5) at a flow rate of 0.8 mL/min. Detection was carried out at 193 nm for nitrite and 213 nm for nitrate.

### 2.4. Study Design and Procedures

Participants were required to visit the laboratory on three separate occasions. During the first visit, they were familiarized with the testing procedure and equipment, including cognitive, perceptual and mood tests, and body weight (kg) and height (m) were measured using digital scales and a stadiometer, respectively. Participants were instructed to complete a food diary for two days prior to the second study visit and to replicate their diet and lifestyle factors for the third study visit (dietary nitrate was not restricted, in an attempt to investigate additional benefits of BR in conjunction with normal daily intake; however, the food intake diary was used to evaluate intake of dietary nitrate and correlated to baseline plasma nitrate and nitrite concentrations). Participants were asked to arrive at the laboratory in a well-rested, fasted state and to refrain from excessive exercise and alcohol consumption 24 h prior and caffeine 6 h prior to each study visit. All trials were performed in the morning at approximately the same time of day (±2 h). 

The second and third visits were the supplementation trials, with each participant randomly allocated (using a random number generator) the order of consumption of the two beverages in a double-blind, crossover design. For these two study visits, participants consumed 150 mL of beetroot juice (BR; containing 10.5 mmol NO_3_^−^) during one visit and 150 mL of a placebo (PL; containing 1 mmol NO_3_^−^), on the other visit. A 150-mL beverage was used to obtain approximately 10.5 mmol of nitrate as this amount has been shown to be a sufficient dose to elicit beneficial effects [4]. Drinks were consumed with a standardized, isocaloric breakfast (cereal or toast) with the same macronutrient breakdown (15 g protein, 30 g carbohydrates and 10 g fat). Participants consumed the same breakfast type for both supplementation trials. Visits two and three were separated by a one-week washout period providing sufficient time for normalization of NO_3_^−^ and NO_2_^−^ levels in the body [8].

**Figure 1:**
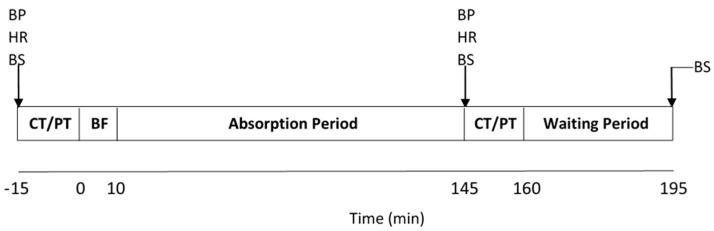
Schematic of the study protocol. CT, cognitive tests; PT, perceptual tests; BF, breakfast; BP, blood pressure; BS, blood sample; HR, heart rate.

The protocol for visits two and three is illustrated in Figure 1. Prior to consumption of the beverage, baseline measures of blood pressure (mean value of three measurements taken from the left arm: deluxe HEM-7130; OMRON Healthcare CO. Ltd.; Kyoto, Japan), heart rate (A1 polar chest transducer), cognitive function, perceptual and mood tests were completed followed by collection of a resting blood sample. Participants then consumed the allocated drink with breakfast, within a 10-min period. Participants were asked to remain in the laboratory for a 2.25 h absorption period while a home development show was aired for entertainment purposes (has minimal effect on cognitive stimulation or arousal levels [4]), or participants could complete computer work or study. The 2.25 h absorption period was chosen as previous research has shown that peak plasma nitrite concentrations occur 2–3 h after beetroot juice consumption [18]. After 2.25 h, the aforementioned measurements were repeated. Blood samples were also taken again 3.25 h post-supplementation. 

### 2.5. Blood Measurements

Plasma nitrite and nitrate were used as biomarkers for nitric oxide availability [19]. Six milliliter venous blood samples were taken by venepuncture, from a vein within the antecubital area, and collected into heparinized tubes. Samples were mixed, centrifuged (MF–50 Hanil Science Industrial, Incheon, South Korea) at 3500 rpm (1330 g) for 10 min, and the collected plasma aliquoted into Eppendorf tubes (0.5 mL per tube) and stored at −80 °C for later analysis of nitrite and nitrate concentrations by HPLC [20].

### 2.6. Cognitive Measurements

Cognitive tests were completed in a quiet, isolated room using a computer placed at eye level (PsychoPy software, version 1.83.04 [21]). The cognitive tasks included the choice reaction test (CRT), rapid visual information processing (RVIP), and Stroop tests. Each task was performed a total of four times per participant (two per trial; pre- and 2.5 h post-supplementation). These tests have been used to investigate the effects of nutritional supplementation on executive function, attention, and information processing speed [4,22].

#### 2.6.1. Choice Reaction Test (CRT)

The CRT task involves participants pressing the left-hand key when the word ‘LEFT’ appears and the right-hand key when the word ‘RIGHT’ appears. 

#### 2.6.2. Rapid Visual Information Processing (RVIP)

The RVIP test involves participants following a chain of single numbers from 1 to 9, which appear in a pseudo-random order at the rate of 100 per minute. The aim of the test is to correctly identify target sequences (2–4–6, 4–6–8, and 3–5–7) as quickly as possible. 

#### 2.6.3. Stroop Test

The Stroop test involves the display of different color names (‘RED’, ‘GREEN’, and ‘BLUE’) which appear on the screen, one at a time in different colored fonts (red, green or blue). The aim of this test is to press the key corresponding to the color of the font that the present word was displayed in. 

### 2.7. Mood and Perceptual Measurements

The feeling scale (FS) was used to measure the degree of displeasure or pleasure based on an 11-point scale ranging from −5 (very bad) to +5 (very good) [23]. The felt arousal scale (FAS) was used to measure the degree of arousal-activation based on a 6-point scale ranging from 1 (low arousal) to 6 (high arousal) [24]. The profile of mood states (POMS) was used to evaluate mood based on ranking how one feels, on a 5-point Likert scale ranging from 0 (not at all) to 4 (extremely) for each word presented under the 7 mood states (fatigue, anger, vigor, tension, esteem, confusion, and depression) [25]. The FS, FAS and POMS measures were all completed before and 2.5 h post-supplementation.

### 2.8. Statistical Analysis

Statistical analyses were completed using IBM SPSS (Version 22.0). All data except for physical characteristics and baseline measurements were analyzed using mixed-method repeated-measures analysis of variance (ANOVA) with treatment and time as within-subject factors, and age as the between-subject factor. Sphericity was tested using the Mauchly’s test to ensure the assumption of sphericity was not violated, and multivariate models were applied if these assumptions were not met. Where significant differences were found, post-hoc tests with Holm-Bonferroni correction were undertaken to assess multiple comparisons. Outliers were excluded based on the Tukey test. Relative effect sizes were calculated using partial eta squared and defined as small (η^2^*_p_* = 0.01), medium (η^2^*_p_* = 0.06), or large (η^2^*_p_* = 0.14). Independent Student’s *t*-tests were used to examine differences in physical characteristics (height, weight, age) between age groups. Data are presented as mean ± standard deviation. Statistical significance was set at *p* < 0.05.

## 3. Results

### 3.1. Participants

The physical characteristics and baseline nitrate, nitrite and blood pressure measurements for the younger and older group participants are reported in Table 1. All 24 participants completed the blood pressure, mood, perceptual and cognitive tests, and treadmill walking exercise. However, blood samples were only successfully obtained from 19 of the 24 (11 younger and 8 older) participants. One older participant was excluded from the Stroop test as their result was determined to be an outlier (using Tukey test) falling outside the interquartile range. 

### 3.2. Plasma Nitrite and Nitrate 

**Figure 2:**
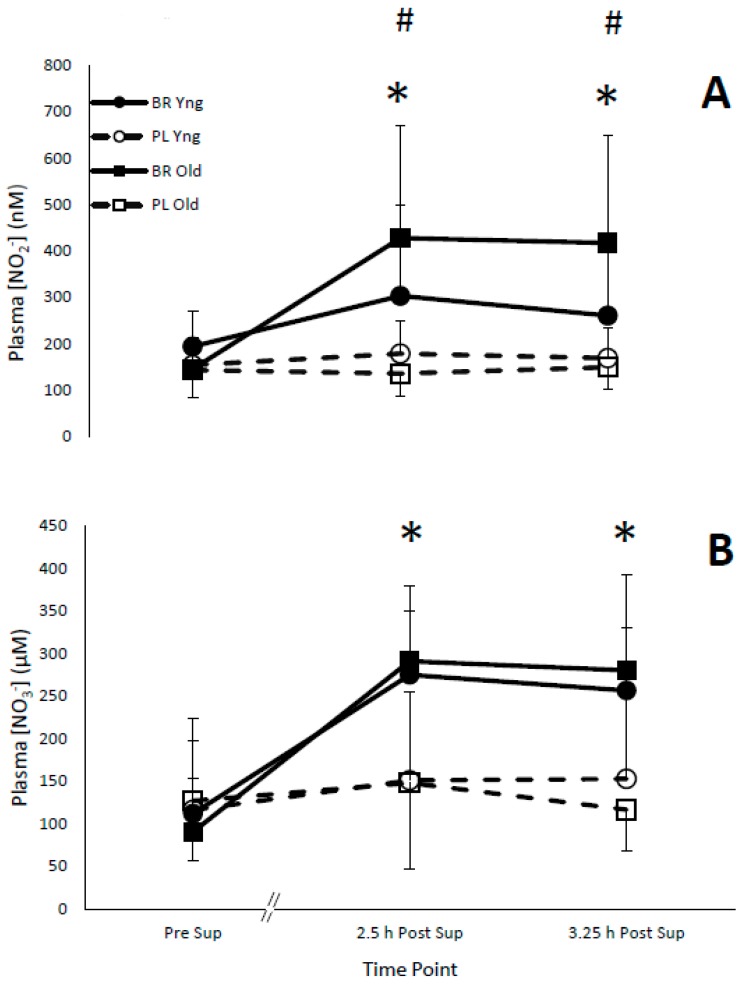
(**A**) Plasma nitrite concentration ([NO_2_^−^]) (nM) pre- (Pre Sup), post-supplementation (Post Sup) and post-exercise (Post Ex) with beetroot juice (BR) and placebo (PL) in younger (Yng) (*n* = 13) and older (Old) adults (*n* = 11). (**B**) Plasma nitrate concentration ([NO_3_^−^]) (µM) pre- (Pre Sup), post-supplementation (Post Sup) and post-exercise (Post Ex) with beetroot juice (BR) and placebo (PL) in younger (Yng) and older (Old) adults. Values are expressed as means ± SD. * Significant mean difference between treatment (placebo vs. beetroot), and time (pre- vs. post-supplementation) (*p* < 0.05). # Significant mean difference between treatment, time and age (*p* < 0.05).

There was no significant difference (*p* < 0.05) between baseline plasma [NO_2_^−^] and [NO_3_^−^] between the groups (Table 1). Plasma [NO_2_^−^] was elevated 2.25 and 3.25 h post-supplementation following BR consumption compared to PL) in both groups (*p* = 0.003, η^2^*_p_* = 0.41, Figure 2A). Older adults had a greater increase in plasma [NO_2_^−^] 2.5 and 3.5 h post-supplementation compared to younger adults following BR supplementation versus PL (*p* = 0.038, η^2^*_p_* = 0.23). Plasma [NO_3_^−^] was also elevated 2.25 and 3.5 h post-supplementation with BR consumption compared to PL in both groups (*p* < 0.001, η^2^*_p_* = 0.67, Figure 2B). There was a trend for a greater increase in plasma [NO_3_^−^] in older adults, 3.5 h post BR supplementation versus PL (*p* = 0.064, η^2^*_p_* = 0.19).

### 3.3. Blood Pressure

**Figure 3:**
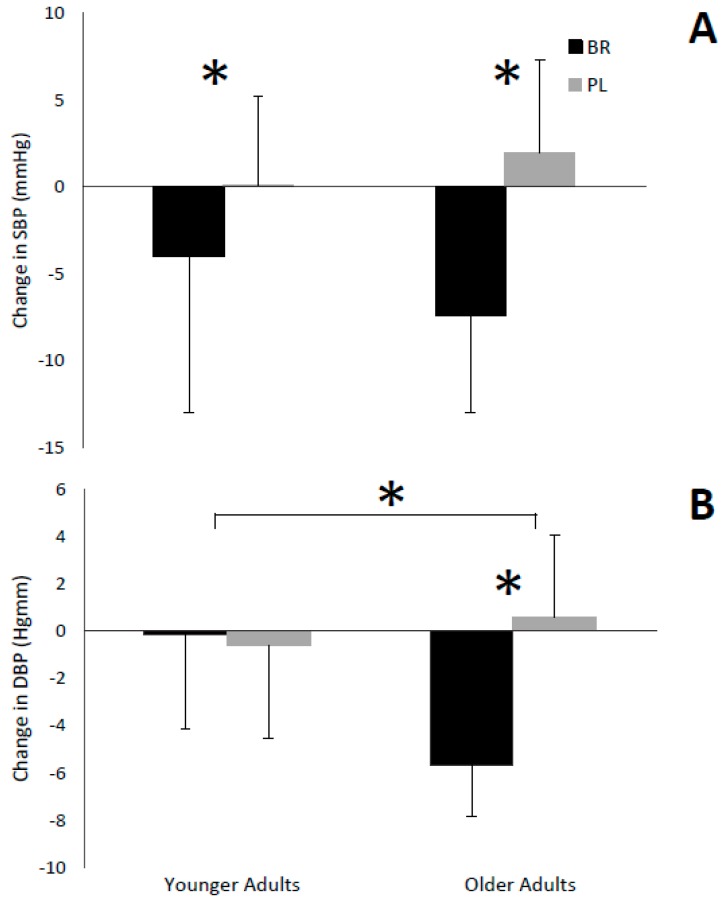
(**A**) Change in systolic blood pressure (SBP) (mmHg) pre- and post-supplementation with beetroot juice (BR) and placebo (PL) in younger (*n* = 13) and older adults (*n* = 11). (**B**) Change in diastolic blood pressure (DBP) (mmHg) pre- and post-supplementation with beetroot juice (BR) and placebo (PL) in younger and older adults. Values are expressed as means ± SD. * Significant mean difference between change in blood pressure (**A**,**B**) and change in blood pressure between older and younger adults (**B**) (*p* < 0.05).

Systolic blood pressure (SBP) was reduced in both younger and older adults following consumption of BR (*p* < 0.001, η^2^*_p_* = 0.45, Figure 3A). However, there was no interaction effect for SBP of treatment*time*age (*p* = 0.11, η^2^*_p_* = 0.11). There was a reduction in DBP post-supplementation compared to pre-supplementation following consumption of BR versus PL for older but not younger adults (*p* = 0.013, η^2^*_p_* = 0.25, Figure 3B). Older adults had a greater reduction in DBP post- versus pre-supplementation with BR versus PL, compared to younger adults (*p* = 0.005, η^2^*_p_* = 0.31, Figure 3B). 

### 3.4. Heart Rate 

There was no interaction of treatment*time (pre- and post-supplementation) (*p* = 0.99, η^2^*_p_* < 0.001) or interaction of treatment*time*age (*p* = 0.62, η^2^*_p_* = 0.011) on average HR in either group. 

### 3.5. Cognitive Performance

Supplementation with BR showed a trend for improved correct response percentage in the Stroop test from pre- to post-supplementation compared to PL (*p* = 0.075, η^2^*_p_* = 0.14). Beetroot juice improved reaction time during the Stroop test from pre- to post-supplementation compared to PL (*p* = 0.045, η^2^*_p_* = 0.18, Table 2). There was no interaction of treatment*time*age for correct responses (*p* = 0.99, η^2^*_p_* < 0.001) or reaction time (*p* = 0.38, η^2^*_p_* = 0.037) in the Stroop test. Placebo improved correct response percentage for CRT from pre- to post-supplementation compared to BR (*p* = 0.008, η^2^*_p_* = 0.28). There was no effect of treatment*time for reaction time in the CRT (*p* = 0.14, η^2^*_p_* = 0.094) or interaction effect of treatment*time and age for percentage correct responses (*p* = 0.96, η^2^*_p_* < 0.001) or reaction time (*p* = 0.076, η^2^*_p_* = 0.14) in the CRT (*p* = 0.96, η^2^*_p_* < 0.001). There were no interaction effects of treatment*time or treatment*time*age for RVIP percentage correct responses (*p* = 0.33 and 0.89, η^2^*_p_* = 0.043 and 0.001, respectively), percentage of errors (*p* = 0.92 and 0.65, η^2^*_p_* < 0.001 and 0.010, respectively), and reaction time (*p* = 0.31 and 0.99, η^2^*_p_* = 0.047 and <0.001, respectively). 

### 3.6. Perceptual Responses and Mood

There was no treatment or treatment*age effects between pre- and post-supplementation for ratings of pleasure-displeasure (FS) (*p* = 0.93 and 0.73, η^2^*_p_* < 0.001 and 0.006, respectively; Table 2) and perceived activation (FAS) (*p* = 0.12 and 0.78, η^2^*_p_* = 0.11 and 0.003 respectively; Table 2). There was no treatment or treatment*age effects for individual categories (*p* > 0.05) or total POMS score (*p* = 0.62 and 0.077, η^2^*_p_* = 0.012 and 0.14, respectively) between pre- and post-supplementation.

## 4. Discussion

We examined the effects of acute supplementation of NO_3_^−^ rich beetroot juice (BR) on cardiovascular responses, cognition, mood and perceptual responses in younger and older adults. The main findings of this study demonstrate that consumption of NO_3_^−^ rich BR; (1) significantly elevated plasma NO_2_^−^ and NO_3_^−^ concentrations and reduced BP for both younger and older adults, (2) improved reaction time during the Stroop test pre- versus post-supplementation, and (3) elevated plasma NO_2_^−^ to a greater degree and resulted in a greater reduction in DBP in older adults.

### 4.1. Plasma [NO_2_^−^] and [NO_3_^−^]

This is the first study to directly compare plasma [NO_2_^−^] and [NO_3_^−^] in younger and older adults following BR supplementation, with consumption of BR significantly elevating both plasma [NO_2_^−^] and [NO_3_^−^] in both age groups. Previous studies have found similar increases in plasma [NO_2_^−^] and [NO_3_^−^] following dietary NO_3_^−^ supplementation in older [15] and younger adults [26]. Supplementation with BR resulted in a greater increase in plasma [NO_2_^−^] and showed a trend for a greater increase in plasma [NO_3_^−^] in older compared to younger adults (Figure 2). This finding was expected as older adults (60–70 years) have been suggested to have an age-related decrease in NO activity, suggesting that NO_3_^−^ supplementation may have a greater impact on the older population, resulting in larger increases in plasma [NO_2_^−^] and [NO_3_^−^] [4]. Due to the novelty of this finding, further research is required directly comparing plasma [NO_2_^−^] and [NO_3_^−^] in younger and older adults following BR supplementation. 

### 4.2. Blood Pressure

Systolic blood pressure was reduced in both age groups following BR supplementation, while DBP was reduced only in the older adults. Studies have shown that reductions in SBP and DBP of as little as 2 mmHg can greatly reduce the incidence of CVD in both hypertensive and normotensive individuals [27]. Consequently, reductions such as those observed in this study are considered clinically meaningful [28]. 

The reductions in BP observed in this study were likely due to an increase in NO production [8], which has been shown to activate the enzyme guanylate cyclase which catalyzes the conversion of guanosine triphosphate (GTP) to cyclic guanosine monophosphate (cGMP) [8,29]. cGMP acts on smooth muscle causing relaxation, resulting in vasodilation of arteries and veins, thus decreasing BP [4]. It would be expected that NO_3_^−^ supplementation would also reduce DBP via this same mechanism; however, this only occurred in older adults. Previous studies have indicated that a reduction in DBP in younger adults following BR consumption is less common compared to a reduction in SBP [8,30], while studies in older adults more commonly show reductions in DBP following BR supplementation [4,15]. Although it has been suggested that the BP-lowering effects of BR may be related to other bioactive compounds or micronutrients found in beetroot juice; studies using a nitrate depleted placebo (by ion-exchange chromatography), and thus containing all other compounds, have still shown similar reductions in BP, indicating the nitrate content is the determining factor [8,9]. 

Interestingly, older adults were found to have a larger reduction in DBP following supplementation with BR compared to younger adults; however, this was not seen for SBP. The interaction effect on DBP is a relevant finding as older adults are at a higher risk of endothelial dysfunction and cardiovascular disease, of which high blood pressure is a major risk factor, due to age-related reductions in NO production [2]. This finding also supports the theory that older adults have a lower initial level of NO and reduced endothelial functioning capacity, thus NO_3_^−^ supplementation may have a greater potential for reducing BP in older versus younger adults [4,16]. In contrast to our findings, one study comparing the effects of dietary NO_3_^−^ supplementation in younger versus older adults found no significant difference in SBP or DBP between the two age groups [16], however, they did not use a placebo-controlled, crossover design which could have impacted the results.

### 4.3. Cognitive Performance

Current findings suggest only certain aspects of cognitive performance (Stroop but not CRT, RVIP) were improved following BR supplementation. This is consistent with the findings of others [22], which showed significant improvements in Stroop test mean reaction time post BR (12.8 mmol of NO_3_^−^) consumption compared to PL (0.08 mmol of NO_3_^−^), but no changes in other cognitive performance tests. Supplementation with NO_3_^−^ rich BR has been shown to significantly increase cerebral perfusion, specifically to the prefrontal cortex [13], therefore, improvements in reaction time in the Stoop test (a task that specifically targets the prefrontal cortex) could be expected. This may be due to increases in cerebral oxygenation allowing for a decrease in the O_2_ cost of mental processing [31]. The variation in results from the different cognitive tests may be due to differences in the cognitive demands of the tests, with the Stroop test placing greater strain on an individual’s mental processing capacity compared to simple reaction and number recall tests. Thus, BR supplementation may only improve cognitive performance when a large degree of cognitive difficulty is imposed [22]. It is also important to note that placebo supplementation improved the correct response percentage for the CRT, however, due to the extremely high percentages of correct responses and small standard deviations, it is possible this result lacks biological significance and indicates the need for further investigation.

While it has been proposed that supplementation with NO_3_^−^ rich BR could have greater benefits on cognitive function in older adults due to its ability to compensate for age-related decreases in NO production and CBF [13], our findings did not support this. The lack of difference between age groups may have been due to the small sample size used and slightly younger mean age (56 years) of the older adults, as results from the CRT showed a trend for greater reduction in reaction time for older adults following BR supplementation (*p* = 0.076). Another major limitation in this area was the lack of a direct measure of CBF, thus it was not possible to determine whether or not BR supplementation increased blood flow to the brain and whether any increase was greater in the older adults. 

### 4.4. Mood and Perception 

Supplementation with NO_3_^−^ rich BR did not improve any mood or perceptual measures (Table 2), supporting the findings of others [31]. Mood is affected by changes in blood flow to the brain and increases in neuro-excitation [32], thus, supplementation with dietary NO_3_^−^ has been suggested as a mood enhancer due to its effects on cerebral vasodilation and stimulation of NO production [6,14]. Studies have also shown that age-related depression and alterations in mood are related to a reduction in CBF, and changes in neurotransmitter concentrations [32]. As BR supplementation increases CBF and stimulates increased NO production, it is possible that older adults may experience greater improvements in mood following consumption [4,13]. The lack of change in mood in this study could be due to insufficient sample size. In addition, it has been shown that age-related depression tends to occur in the mid-to-late 60s, [32] hence the lack of significance in mood and perception values between age groups seen here may have been due to the mean age of the older group being only 56 y.

### 4.5. Future Directions

The current findings indicate the potential for NO_3_^−^ rich BR as an intervention for reducing BP in normotensive younger and older adults, with further research needed to assess its viability in a medical setting; for example, being used in conjunction with antihypertensive medication or to help delay the need for pharmaceutical treatment in populations with hypertension. The findings also highlight the need for further research, with specific attention to direct comparisons of measures in younger and older adults, larger sample size for cognitive and mood measures, and more sensitive and specific measures for potential cognitive and mood benefits from supplementation with NO_3_^−^ rich BR (e.g., near-infrared spectroscopy (NIRS) of the brain to monitor oxygenation and CBF [15]). Additionally, as this study showed a benefit on BP following NO_3_^−^ rich BR supplementation in the healthy population this may suggest greater benefits in hypertensive groups, which should be an area for future research. Moreover, further research is needed to examine the effects of long-term consumption (chronic) of NO_3_^−^ rich BR supplementation on health benefits. To date, most longer-term studies, involve daily consumption for periods of between 6 to 14 days [9,12] so future studies should focus on investigating longer supplementation periods of at least 28 days duration. 

## 5. Conclusions

Acute supplementation with NO_3_^−^ rich BR increased plasma [NO_2_^−^] and [NO_3_^−^] and reduced SBP in both age groups. The increase in plasma NO_2_^−^ was greater in older adults, and DBP was reduced in older compared to younger adults. There was no difference in HR. Reaction time was improved in the Stroop test following BR supplementation; however, no difference was seen between age groups or for any other cognitive tests. Supplementation with BR did not improve perceptual or mood measures pre- versus post-supplementation. Collectively, these results indicate that acute supplementation with BR can reduce BP and improve aspects of cognitive performance; thus having potential health benefits for both younger and older adults.

## Figures and Tables

**Table 1:** Physical characteristics and baseline nitrate, nitrite and blood pressure measurements of younger and older adult group participants. ^1^

	**Younger Adults (*n* = 13)**		**Older Adults (*n* = 11)**		***p*-Value**	
Male	9		3			
Female	4		8			
Age (years)	25 ± 3		56 ± 6		<0.001 *	
Height (cm)	178.4 ± 10.1		169.8 ± 7.0		0.026 *	
Body Mass (kg)	75.3 ± 14.9		74.7 ± 7.9		0.90	
	**Beetroot**	**Placebo**	**Beetroot**	**Placebo**	**Beetroot (Between Groups)**	**Placebo (Between Groups)**
Plasma NO_2_^−^ (nM)	193.9 ± 77.5	153.8 ± 57.7	145.4 ± 40.2	143.1 ± 60.3	0.19	0.86
Plasma NO_3_^−^ (μM)	111.8 ± 112.5	116.0 ± 80.9	90.3 ± 62.1	127.0 ± 71.1	0.90	0.27
SBP (mmHg)	116.8 ± 8.1	114.9 ± 9.2	125.2 ± 9.6	120.9 ± 10.8	0.061	0.003 *
DBP (mmHg)	68.5 ± 6.0	68.8 ± 7.3	80.9 ± 6.9	77.9 ± 6.3	0.81	0.042 *

^1^ Values are means ± SD. * indicates significant difference (*p* < 0.05).

**Table 2:** Cognitive performance tests and mood analysis pre- and post-supplementation with beetroot juice and placebo in younger and older adults. ^1^

	Younger Adults				Older Adults			
	Placebo		Beetroot		Placebo		Beetroot	
	*Pre*	*Post*	*Pre*	*Post*	*Pre*	*Post*	*Pre*	*Post*
**Choice Reaction Test**								
Correct Response (%)	96.2 ± 2.3	97.5 ± 1.7 *	96.2 ± 2.1	95.6 ± 2.6	98.6 ± 2.0	99.2 ± 0.4 *	98.7 ± 1.7	97.4 ± 2.2
Reaction Time (ms)	433 ± 48	426 ± 34	435 ± 46	421 ± 45	637 ± 125	588 ± 59	596 ± 96	615 ± 134
**Rapid Visual Information Processing**								
Correct Response (%)	87.2 ± 9.5	90.1 ± 8.8	81.1 ± 16.7	89.5 ± 7.7	57.8 ± 20.3	63.1 ± 21.4	55.9 ± 20.6	65.4 ± 16.9
Errors	1.2 ± 1.2	1.2 ± 1.1	1.4 ± 1.3	1.1 ± 1.1	2.4 ± 3.4	1.3 ± 1.7	2.6 ± 2.0	1.8 ± 1.6
Reaction Time (ms)	422 ± 34	402 ± 32	445 ± 67	409 ± 49	481 ± 65	495 ± 61	498 ± 68	496 ± 76
**Stroop Test**								
Correct Response (%)	96.5 ± 2.6	95.8 ± 3.6	95.6 ± 3.0	96.3 ± 2.8	98.0 ± 2.2	98.6 ± 1.1	96.7 ± 3.0	98.7 ± 1.6
Reaction Time (ms)	644 ± 112	607 ± 102	690 ± 204	616 ± 117 *	965 ± 194	970 ± 167	978 ± 189	891 ± 138 *
**Mood Measures**								
FS	1.9 ± 1.1	2.2 ± 1.1	2.6 ± 1.3	2.9 ± 1.3	2.7 ± 1.2	2.8 ± 1.5	2.2 ± 1.8	2.4 ± 1.3
FAS	1.9 ± 0.8	2.4 ± 1.0	2.2 ± 0.6	2.3 ± 1.0	1.9 ± 0.9	2.0 ± 1.1	2.1 ± 1.2	1.9 ± 1.3
POMS	22 ± 11	17 ± 7	22 ± 9	19 ± 8	18 ± 8	16 ± 7	25 ± 10	20 ± 10

^1^ Values are expressed as means ± SD. Younger (*n* = 13), Older [choice reaction, rapid visual information processing and mood tests (*n* = 11) Stroop test (*n* = 10). * Significant mean difference between treatment (placebo vs. beetroot), and time (pre- vs. post-supplementation) (*p* < 0.05). FS, feeling scale; FAS, felt arousal scale; POMS, profile of mood states.
